# Removal of Foreign Bodies from the Ear Passages

**Published:** 1850-05

**Authors:** 


*Removal of foreign bodies from the ear-passages.*—Mr. Toynbee, in a communication to the Provincial Journal, condemns the use of forceps or curettes, and says—"The instrument that I have always found successful in removing foreign bodies from the external auditory meatus without fear of producing inflammation, is a syringe, of capacity sufficient to hold two or three ounces of water. The plan of using a syringe in these cases has been often recommended, but I feel confident that it will not prove successful unless the syringe be of sufficient size to allow the water to be injected with considerable force. Forceps and other instruments are had recourse to by surgeons who have found that the foreign body is not moved by the use of a small syringe, the great fault of which is that sufficient force cannot be used to cause the water to pass between the foreign substance, when it is large and round, and the walls of the meatus; or, if the substance is heavy, an outward current powerful enough to carry it towards the orifice cannot be induced. Of the foreign bodies which I have recently removed from the external auditory meatus by means of the syringe, two consisted of a pea and a bead, both appearing, on examination with the speculum, to be closely surrounded by the membranous meatus; the third was a piece of slate pencil lying at the lower wall of the inner extremity of the tube, and in contact with the membrana tympani. I feel confident that an attempt to extract any of these substances by means of forceps or director would have been attended by injury to the meatus or membrana tympani, whereas each was removed by the use of a syringe for about three minutes.—*London Medical Gazette.*

